# MF-094 nanodelivery inhibits oral squamous cell carcinoma by targeting USP30

**DOI:** 10.1186/s11658-022-00407-8

**Published:** 2022-12-06

**Authors:** Xinyu Zhang, Yong Han, Shuli Liu, Bing Guo, Shengming Xu, Yue He, Liu Liu

**Affiliations:** 1 Department of Oral and Maxillofacial-Head and Neck Oncology, Shanghai Ninth People’s Hospital, College of Stomatology, Shanghai Jiao Tong University School of Medicine, No.639 Zhizaoju Road, Huangpu District, Shanghai, 200011 China; 2 National Clinical Research Center for Oral Diseases, Shanghai, China; 3 Shanghai Key Laboratory of Stomatology and Shanghai, Research Institute of Stomatology, Shanghai, China; 4 Department of Plastic and Reconstructive Surgery, Shanghai Key Laboratory of Tissue Engineering, Shanghai Ninth People’s Hospital, Shanghai Jiao Tong University School of Medicine, Shanghai, China

**Keywords:** Head and neck cancer, Nanoparticles, ZIF-8, Ubiquitination, c-Myc

## Abstract

**Background:**

Oral squamous cell carcinoma (OSCC) is a common head and neck cancer, and the incidence of OSCC is increasing. As the mortality of OSCC keeps increasing, it is crucial to clarify its pathogenesis and develop new therapeutic strategies.

**Methods:**

Confocal laser scanning microscopy was used to evaluate the uptake of nanoparticles (NPs). The potential functions of USP30 were evaluated by cell counting kit (CCK)-8, flow cytometry, biochemical assay, coimmunoprecipitation, qRT–PCR, and immunoblotting. The antitumor effect of NP-loaded USP30 inhibitor MF-094 was evaluated in vitro and in vivo.

**Results:**

In this study, increased USP30 expression was found in OSCC specimens and cell lines through qRT–PCR and immunoblotting. CCK-8, flow cytometry, and biochemical assay revealed that the deubiquitylated catalytic activity of USP30 contributed to cell viability and glutamine consumption of OSCC. Subsequently, USP30 inhibitor MF-094 was loaded in ZIF-8-PDA and PEGTK to fabricate ZIF-8-PDA-PEGTK nanoparticles, which exhibited excellent inhibition of cell viability and glutamine consumption of OSCC, both in vitro and in vivo.

**Conclusion:**

The results indicated the clinical significance of USP30 and showed that nanocomposites provide a targeted drug delivery system for treating OSCC.

**Supplementary Information:**

The online version contains supplementary material available at 10.1186/s11658-022-00407-8.

## Background

Oral squamous cell carcinoma (OSCC) is a common head and neck cancer. Smoking and alcohol are the most common risk factors of OSCC [1]. The 5-year survival rates remain at less than 50% [2]. Although medical advances have been made, morbidity and mortality have not improved dramatically. Additionally, the incidence of OSCC is increasing among young people [3]. Systemic drug therapy is one of the approaches to treat advanced OSCC, but treatments for advanced OSCC, such as platinum drugs, 5‐fluorouracil, paclitaxel, and doxorubicin, only improve the 5‐year overall survival rate to 50%. Therefore, it is crucial to explore novel biomarkers with prognostic value for patients with OSCC.

Ubiquitination is one of the most common posttranslational modifications in cells and dysregulation is closely associated with the development of cancer [4]. Ubiquitination can be reversed by deubiquitinases (DUBs) [5]. As one of the five classes of DUBs, ubiquitin-specific proteases (USPs) regulate the deubiquitination/ubiquitination process [6]. Dysregulation of the deubiquitination/ubiquitination process has been observed in various diseases including cancers [7]. USP30 is one of only two DUBs that possess a transmembrane domain. Studies demonstrate that USP30 plays a very important role in various biological processes. For instance, USP30 deubiquitinase suppresses mitophagy via opposing ubiquitination [8]. Yan et al. showed that USP30 inhibition promotes apoptosis of lung cancer cells [9]. USP30 suppression sensitizes cancer cells to BH3-mimetics [10]. USP30 can deubiquitinate and stabilize mitochondrial division protein DRP1 and promote mitochondrial morphology, and thus regulate lipid metabolism and the occurrence of liver cancer [11]. However, the biological function of USP30 in OSCC is still unclear.

Only a few DUB inhibitors have entered clinical testing, and none have been approved. The current clinical pipeline includes small molecules targeting USP1 and USP30 [12]. Recently, MF-094, a potent and selective USP30 inhibitor, accelerates diabetic wound healing by inhibiting the NLRP3 inflammasome [13]. However, it shows some disadvantages including high toxicity and the need of a facilitating drug delivery system [14], which makes the task of prioritizing DUBs for human therapeutics more difficult. Therefore, we plan to introduce the nanomaterial delivery system in this study. To reduce the systemic toxic side effects of drugs, nanomaterials can be used as carriers to target the drug delivery to the lesion site. Among the numerous nanomaterials, metal–organic framework (MOF) materials show the characteristics of high drug loading efficiency and low toxicity. Zeolitic imidazolate framework-8 (ZIF-8), a MOF, has been shown to be an ideal drug carrier because it is stable under physiological conditions but it responds quickly under weak acidic environments [15]. Polydopamine (PDA) has been widely utilized to coat various types of nanomaterial MOFs such as ZIF-8, and subsequent carbonization results in hollow nitrogen-doped carbons, which shows greatly improved supercapacitive and electrocatalytic performances [16]. Moreover, polyethylene glycol-thioketal (PEGTK) coating is a reactive oxygen species-responsive (ROS) trait [17]. The moderate concentration of ROS in cancer cells can stimulate the release of drugs from ZIF-8. Thus, the MF-094@ZIF-8-PDA-PEGTK nanoparticle was used to establish a corresponding molecular inhibitor delivery system in this study, which would provide new ideas and strategies for the clinical treatment of OSCC.

## Materials and methods

### Clinical samples

This study included 20 fresh tumor tissues (10 each in stages I/II and III) and adjacent-normal tissues from OSCC patients admitted to Shanghai Ninth People’s Hospital. Human OSCC tissue microarrays were used for measuring USP30 levels by immunohistochemistry (IHC). The protocols for cancer specimen retrieval had the approval from the medical ethics commission of Shanghai Ninth People’s Hospital (no. SH9H-2022-TK84-1, date: 11 February 2021).

### Immunohistochemistry (IHC)

OSCC tissues were fixed in 10% formaldehyde, embedded in paraffin, and sliced into 4 μm sections. The sections were baked in a 60 °C incubator for 1 h, conventionally dewaxed by xylene, dehydrated with gradient alcohol, incubated in 3% H_2_O_2_ (Sigma–Aldrich Chemical Company, St Louis, MO, USA) at 37 °C for 30 min, and washed with phosphate-buffered saline (PBS; Gibco, Invitrogen, Paisley, UK). The sections were then boiled in 10 mM citric acid buffer at 95 °C for 20 min, cooled to room temperature, and washed with PBS. Sections were incubated with anti-USP30 antibody (Abcam; ab235299), followed by HRP-conjugated anti-IgG antibody (Long Island Biotech, China, D-3004) using the standard protocol. IHC results were analyzed by two pathologists and scored based on percentage of positively stained cells. OSCC patients were divided into the low expression (H-score < 50%) group and the high expression (H-score > 50%) group.

### Cell culture

Human oral epithelial cell HOEC (Procell Life Science & Technology Co., Ltd., China; CP-H203) and human OSCC cell lines SCC4 (CRL-1624), SCC9 (CRL-1629), SCC15 (CRL-1623), CAL27 (CRL-2095; all from ATCC, USA), and HSC4 (RIKEN Cell Bank, Tukuba, Japan; RCB1902) were maintained in DMEM with 10% FBS at 37 °C.

### USP30 silencing or overexpression

shRNA targeting USP30 (shUSP30-1, 5′-GCTGCTTGTTGGATGTCTT-3′; shUSP30-2, 5′-CCAGAGTCCTGTTCGATTT-3′) were inserted into pLKO.1. USP30 cDNA was inserted into pLVX-puro. The recombinant plasmids were transfected and amplified in 293 T cells (ATCC; CRL-3216) along with the packaging plasmids psPAX2 and pMD2G to generate lentiviral particles. Transfection was performed using Lipofectamine 2000 (Invitrogen, Carlsbad, CA, USA) according to the manufacturer’s manual. The lentiviral particles were collected 48 h after transfection and used to transduce OSCC cell lines. Blank pLVX-Puro (vector) and pLKO.1-scrambled shRNA (shNC) were used as negative controls for overexpression and knockdown studies, respectively. C-terminally flag-tagged human USP30 DUB-domain cDNA (wild type or the C77S mutant) expression vectors were constructed as previously described [18].

### Preparation of MF-094@ZIF-8

MF-094@ZIF-8 was synthesized as follows: 180 mg 2-methylimidazole (2-MIM) and 20 mg Zn(NO_3_)_2_ 6H_2_O were dispersed in 1 mL methanol. Then, 0.5 mL MF-094 and 2-MIM was mixed. Next, Zn(NO_3_)_2_ 6H_2_O was quickly injected and mixed for 1.5 h. The product was centrifuged at 7000 rpm for 5 min and washed with methanol. MF-094@ZIF-8 was collected.

### Preparation of MF-094@ZIF-8-PDA

The MF-094@ZIF-8 (50 mg) and 3-hydroxytyramine hydrochloride (10 mg) were poured into 50 mL pH 8.5 Tris–HCl buffer and stirred for 3 h. The mixed solution was spun at 10,000 rpm to get MF-094@ZIF-8-PDA nanoparticles (NPs) and washed with deionized water three times and freeze dried.

### Preparation of MF-094@ZIF-8-PDA-PEGTK NPs

The MF-094@ZIF-8-PDA NPs (50 mg/mL) solution and NH_2_-PEG-NH_2_ (50 mg/mL) aqueous solution were mixed in deionized water with 1-ethyl-3-(-3-dimethyl aminopropyl) carbodiimide hydrochloride (EDC, 50 mg/mL) and N-hydroxysuccinimide (NHS, 50 mg/mL) and stirred for another 24 h. The mixture was spun at 10,000 rpm to get MF-094@ZIF-8-PDA-PEGTK NPs and washed with deionized water three times.

### NP characterization

NPs were observed by a transmission electron microscopy (TEM). The Brunauer–Emmett–Teller (BET) method was used for evaluating surface areas. DynaPro NanoStar (Wyatt, USA) was used to measure the size distribution of NPs. Thermogravimetric analysis (TGA) was used for evaluating the loading capacity of NPs. The chemical composition of NPs was examined with Fourier transform infrared (FT-IR).

### Cellular uptake behavior

We used confocal laser scanning microscopy (CLSM) to evaluate the cellular uptake ability of MF-094@ZIF-8-PDA-PEGTK by HSC4 cells. Briefly, HSC4 cells were seeded in a 24-well plate at a density of 3 × 10^4^ cells/well and incubated with 50 μg/mL of Cy7-labeled MF-094@ZIF-8-PDA-PEGTK. After incubation for 6 h, the nuclei, cytoskeleton, and MF-094@ZIF-8-PDA-PEGTK was stained with 4′,6-diamidino-2-phenylindole (DAPI; Beyotime Biotechnology, Shanghai, China), fluorescein isothiocyanate (FITC)-phalloidin (Abcam), and Cy7 (MedChemExpress) for 40 min, respectively, and observed via CLSM at 405, 562, and 488 nm laser excitation. TEM detection for samples fixed with 4% glutaraldehyde was performed as previously described [19].

### Quantitative RT–PCR (qRT–PCR)

RNA was isolated using TRIzol (Invitrogen, Carlsbad, CA, USA; 15596018) and reverse transcribed into cDNA using the RevertAid First Strand cDNA Synthesis Kit (Thermo Fisher Scientific, Waltham, MA, USA; K1622). The qRT–PCR was performed using a Maxima SYBR Green/ROX qPCR Master Mix (Thermo Fisher Scientific, Waltham, MA, USA; K0223). The primers sequences were as follows: USP30, 5′-CCCAGACAGAACAGAAACAG-3′ and 5′-AAGAGCAGCAGCAATTCC-3′; c-Myc, 5′-AGGCTGCGGCTTGGATTAAC-3′ and 5′-CAAACCCTCTCCCTTTCTC-3′; GAPDH, 5′-AATCCCATCACCATCTTC-3′ and 5′-AGGCTGTTGTCATACTTC-3′. Reaction conditions were: 95 °C for 10 min, followed by 40 cycles of 95 °C for 15 s, 60 °C for 45 s. The fold change was calculated by the 2^−△△CT^ method.

### Immunoblotting

Proteins were isolated with RIPA buffer and the protein concentrations were quantified using a BCA Protein Assay Kit (Beyotime Biotechnology, Shanghai, China; P0012S). Proteins were separated with SDS-PAGE, electro-blotted to membranes, blocked, and probed with anti-USP30 (ab235299), anti-c-Myc (ab32072), anti-GLS1 (ab131554), anti-SLC1A5 (ab187692), and anti-β-actin antibodies (ab8227; all from Abcam) in accordance with the manufacturer’s instruction. After incubating with primary antibodies, the membranes were incubated with HRP-conjugated secondary antibodies (Beyotime Biotechnology, Shanghai, China; A0208, A0216). Chemiluminescence (Merck Chemicals, Co., Ltd, Shanghai, China; WBKLS0100) was used for detecting antibody binding. Image J analysis software was used to quantify the gray levels of each band for protein study. β-Actin served as a loading control.

### Cell viability

Cell viability was measured using cell counting kits (CCK)-8 (Dojindo Molecular Technologies, Kumamoto, Japan; CK04). Briefly, cells were grown to the logarithmic phase, harvested, and seeded into 96-well plates at a cell density of 3 × 10^3^ cells per well. At 12, 24, and 48 h after treatment, 10 μL CCK-8 solution was added to each well and maintained for the reaction time of 1 h. Absorbance was measured at 450 nm using a microplate reader and used for the calculation of cell viability.

### Apoptosis assay

Cells were kept in a 6-well plate (3 × 10^5^/ well) until 50% confluence. After treatment, the cells were collected and incubated with 5 µL of recombinant annexin V labeled with fluorescein isothiocyanate (Annexin V-FITC) for 15 min in the dark at 4 °C, and then with 5 µL Propidium iodide (PI) (Beyotime Institute of Biotechnology) for another 15 min. Apoptosis was examined using a FACScan flow cytometer (BD, Franklin Lakes, NJ, USA).

### Glutamine assay

After treatment, the levels of glutamine in cells were analyzed with a glutamine assay kit (Abcam; ab197011) following the manufacturers’ instructions. According to the principle of glutamine conversion into glutamic acid and ammonia, the amount of glutamine was calculated by measuring the amount of ammonia. The relative glutamine uptake was normalized by the protein amount of each group.

### Co-immunoprecipitation (Co-IP)

Cell lysates were prepared with lysis buffer (1% Triton X-100, 150 mM NaCl, 20 mM Tris pH7.5, and 1 mM EDTA) supplemented with protease inhibitor cocktail (Sigma-Aldrich), incubated with normal immunoglobulin (Ig)G (Santa Cruz Biotechnology; sc-2027), anti-USP30 (Invitrogen; PA5-113053), or anti-c-Myc (Abcam; ab168727) antibodies, followed by further incubation with Protein A/G PLUS-Agarose beads (Santa Cruz Biotechnology; sc-2003) at 4 °C for 2 h. Then, the immunocomplex was washed three times by the lysis buffer for Western blot analysis.

### Half-life of c-Myc

HSC4 cells were infected with lentivirus expressing shUSP30 or control shRNA (shNC) for 24 h, then exposed to cycloheximide (CHX, 20 mM, Sigma-Aldrich). Cells were collected at 0, 1, 4, or 8 h after exposure and subjected to immunoblotting.

### Animal model

Male nude mice (4 weeks old) were subcutaneously injected with 1 × 10^6^ SCC4 cells. Ten days after inoculation, MF-094 or Cy7-labeled NPs loaded with or without MF-094 were injected into the mice via the tail vein, at a dose of 1 mg/kg/day for 4 weeks. Herein, four experiment groups were established (*n* = 10 mice per group), including mice bearing SCC4 cells as control (group 1), mice bearing SCC4 cells injected with MF-094 (group 2), and mice bearing SCC4 cells injected with NPs loaded with (group 3) or without MF-094 (group 4). NP fluorescent images were obtained using the IVIS 200 system (PerkinElmer, Waltham, MA, USA) at 6, 12, and 24 h to verify the NP biodistribution. In vivo surviving SCC4 cells were detected using bioluminescence at 0, 1, 2, 3, and 4 weeks to verify the in vivo targeting efficacy of NPs. Tumor volume was recorded every 4 days. Mice were euthanized on day 38, and their key organs (liver, heart, lungs, kidneys, and spleen) and xenograft tumors were collected, washed, fixed with 4% paraformaldehyde (PFA), and processed to produce paraffin-embedded sections. To evaluate toxicity and apoptosis, sections were stained with H&E or TUNEL. Animal experiments were performed in concordance with the NIH Guidelines and approved protocols of Animal Care and Use Committee of Shanghai Rat@Mouse Biotech Co., Ltd., China (no. 2020-0213, date: 15 March 2020).

### Statistical analysis

Data are presented as the mean ± standard deviation (SD) from at least three independent experiments. Statistical analysis was performed using GraphPad Prism 8.4.2 (San Diego, CA). Student’s *t*-test and ANOVA were applied for comparisons. *P*-values < 0.05 were defined significant.

## Results

### USP30 was upregulated in human OSCC tissue and was associated with poor prognosis

**Fig. 1:**
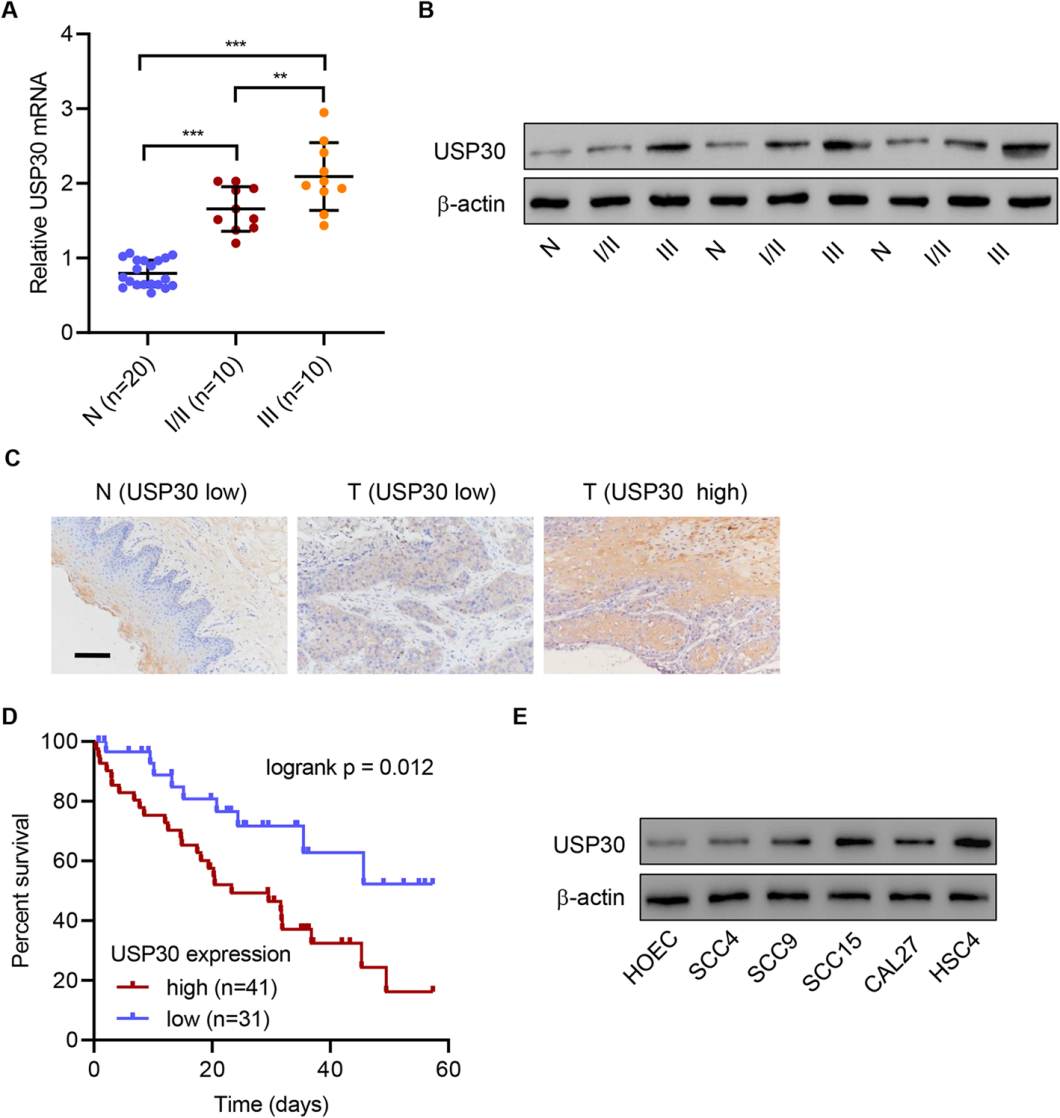
USP30 upregulation is associated with poor prognosis. **A** mRNA and **B** protein levels of USP30 in stage 1 or 2 OSCC tissues (I/II), stage 3 OSCC tissues (III), and adjacent nontumor tissues (N). **C** USP30 protein levels in human OSCC tissue microarrays detected by IHC (scale bars: 100 μm). **D** Overall survival rate analysis. **E** USP30 expression in human OSCC cell lines and oral epithelial cell HOEC. **P < 0.01, ***P < 0.001

To analyze the clinical relevance of USP30, the mRNA levels of USP30 in human OSCC tissue and adjacent normal tissue were measured. qRT–PCR showed that the mRNA level of USP30 was significantly increased in human OSCC tissues compared with adjacent normal tissues (Fig. 1A). The mRNA level was also correlated with the severity of OSCC: tissues from stage III patients showed higher expression of USP30 than those from stage I/II patients. Consistently, Western blot showed that the protein level of USP30 was increased in OSCC tissues, especially in tissue samples from stage III patients (Fig. 1B). Immunohistochemistry analysis confirmed the upregulation of USP30 in tumor tissues and categorized tissues into USP30 high-expression group or USP30 low-expression group (Fig. 1C). The clinicopathological characteristics and follow-up data of OSCC patients with low or high expression of USP30 were shown in Additional file 1: Table S1. Higher USP30 expression was associated with poorer survival rate (Fig. 1D). The expression level of USP30 was measured in different human OSCC cell lines and the results showed that HSC4 cells has the highest USP30 level, and SCC4 and HOEC have very low USP30 levels (Fig. 1E). Therefore, HSC4, SCC4, and HOEC were chosen in this study. These results indicated that USP30 upregulation is associated with poor prognosis.

### Deubiquitylated catalytic activity of wild-type USP30 accounted for increased cell viability and glutamine consumption, and decreased apoptosis in SCC4 cells

**Fig. 2:**
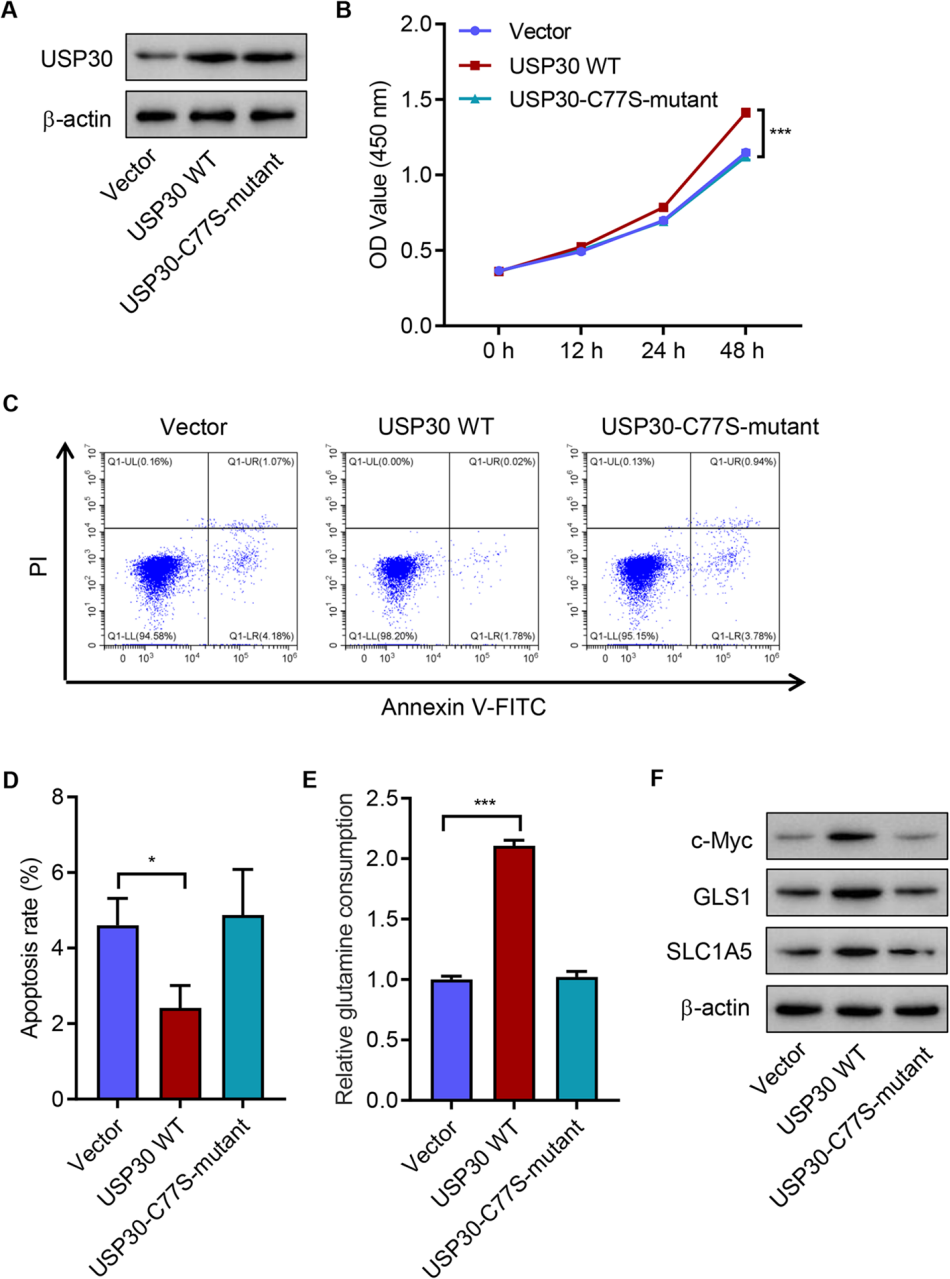
USP30 overexpression increased SCC4 cell viability and glutamine consumption, and inhibits apoptosis. SCC4 cells were transfected with WT-USP30, C77S-mutant USP30, or vector, and (**A**) USP30 expression, (**B**) cell viability, (**C**, **D**) apoptosis, (**E**) glutamine consumption, and (**F**) expression of c-Myc, GLS1, and SLC1A5 were measured. *P < 0.05, ***P < 0.001

To further investigate USP30 function, wild-type (WT)- or C77S-mutant USP30 was overexpressed in SCC4 cells (Fig. 2A). Overexpression of WT-USP30 significantly increased cell viability, decreased cell apoptosis, and increased glutamine consumption compared with the vector control (Fig. 2B–E). Western blotting showed that overexpression of WT-USP30 significantly promoted c-Myc, GLS1, and SLC1A5 expression (Fig. 2F). In contrast, overexpression of C77S-mutant USP30 did not affect SCC4 cell viability, apoptosis, glutamine consumption, or c-Myc, GLS1, and SLC1A5 expression (Fig. 2B–F), suggesting that deubiquitylated catalytic activity plays a key role in exerting the function of USP30. Compared with control cells, silencing USP30 also significantly inhibited the viability, promoted apoptosis, and suppressed glutamine consumption and c-Myc, GLS1, and SLC1A5 expression of HSC4 cells (Additional file 1: Fig S1A–E). The results indicated that USP30 promotes cell viability and glutamine consumption but inhibits apoptosis of OSCC cells.

### USP30 regulates SCC4 cell viability, glutamine consumption, and apoptosis by inducing c-Myc deubiquitination

**Fig. 3:**
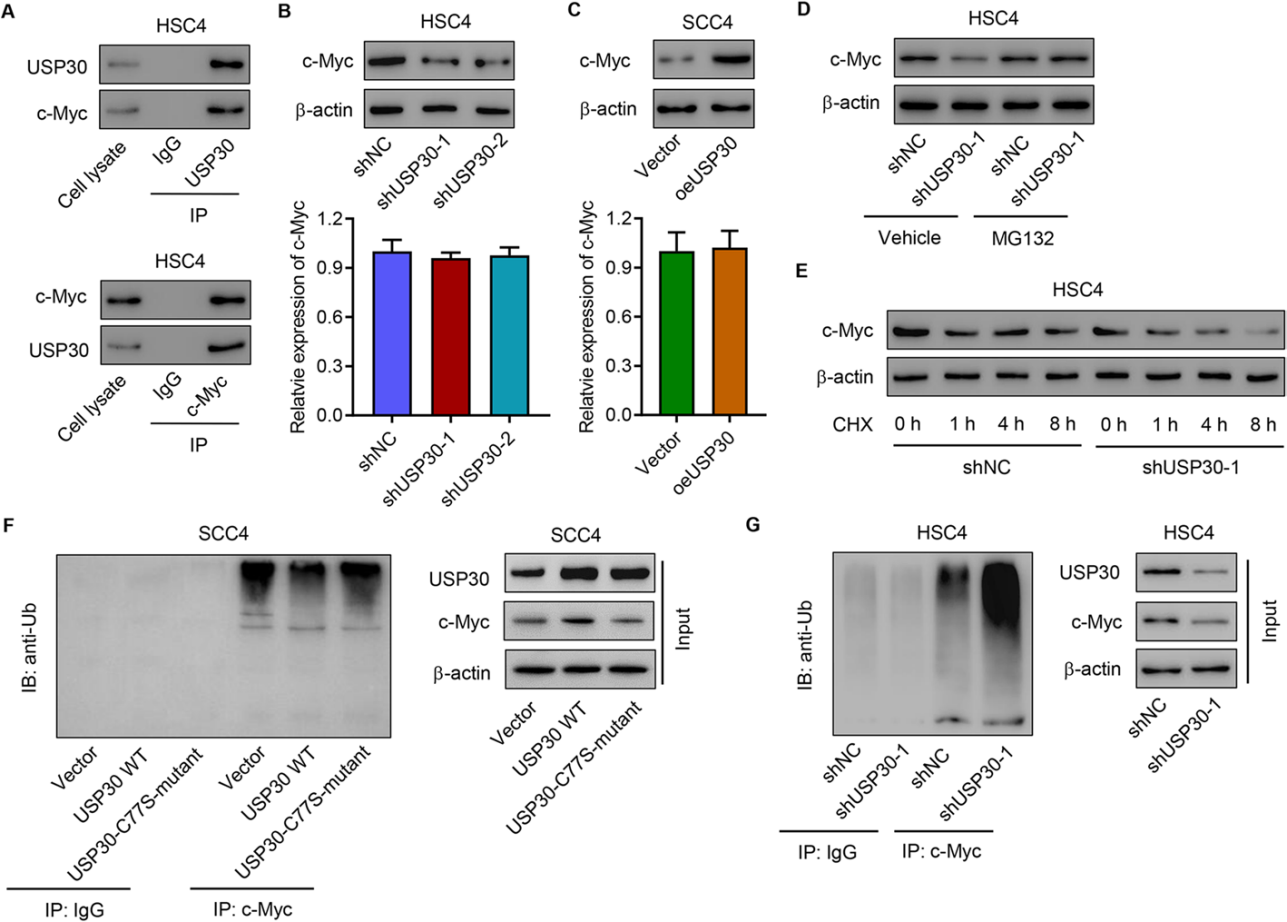
USP30 promotes c-Myc deubiquitination. **A** Interaction between USP30 and c-Myc in HSC4 cells. c-Myc expression in **B** HSC4 and **C** SCC4 cells with or without USP30 knockdown and overexpression. c-Myc levels in HSC4 cells with USP30 knockdown with or without **D** protease inhibitor MG132 (10 μM) or **E** CHX treatment. **F** Effect of USP30 overexpression on c-Myc ubiquitination in SCC4 cells transfected with WT-USP30, C77S-mutant USP30, or vector. **G** Effect of USP30 knockdown on c-Myc ubiquitination in HSC4 cells

To elucidate how USP30 was involved in regulating the viability and apoptosis of OSCC cells, we first determined USP30 binding proteins using co-IP analysis. Figure 3A confirmed the interaction between USP30 and c-Myc. Then, c-Myc was decreased in HSC4 cells or increased in SCC4 cells (Fig. 3B, C). Western blotting results also suggested that at the protein level, USP30 silencing significantly suppressed c-Myc expression, which was abolished by administration of a proteasome inhibitor (MG132) (Fig. 3D). USP30 silencing also significantly promoted the degradation of c-Myc (Fig. 3E). IP analysis of the ubiquitination of c-Myc indicated that overexpressing WT-USP30 significantly decreased the ubiquitination of c-Myc, but C77S-mutant USP30 overexpression showed no effect on the ubiquitination of c-Myc in SCC4 cells (Fig. 3F). Meanwhile, silencing USP30 also promoted c-Myc ubiquitination in HSC4 cells (Fig. 3G). Together, these finding indicated that USP30 interacts with and inhibits c-Myc ubiquitination in OSCC cells.

**Fig. 4:**
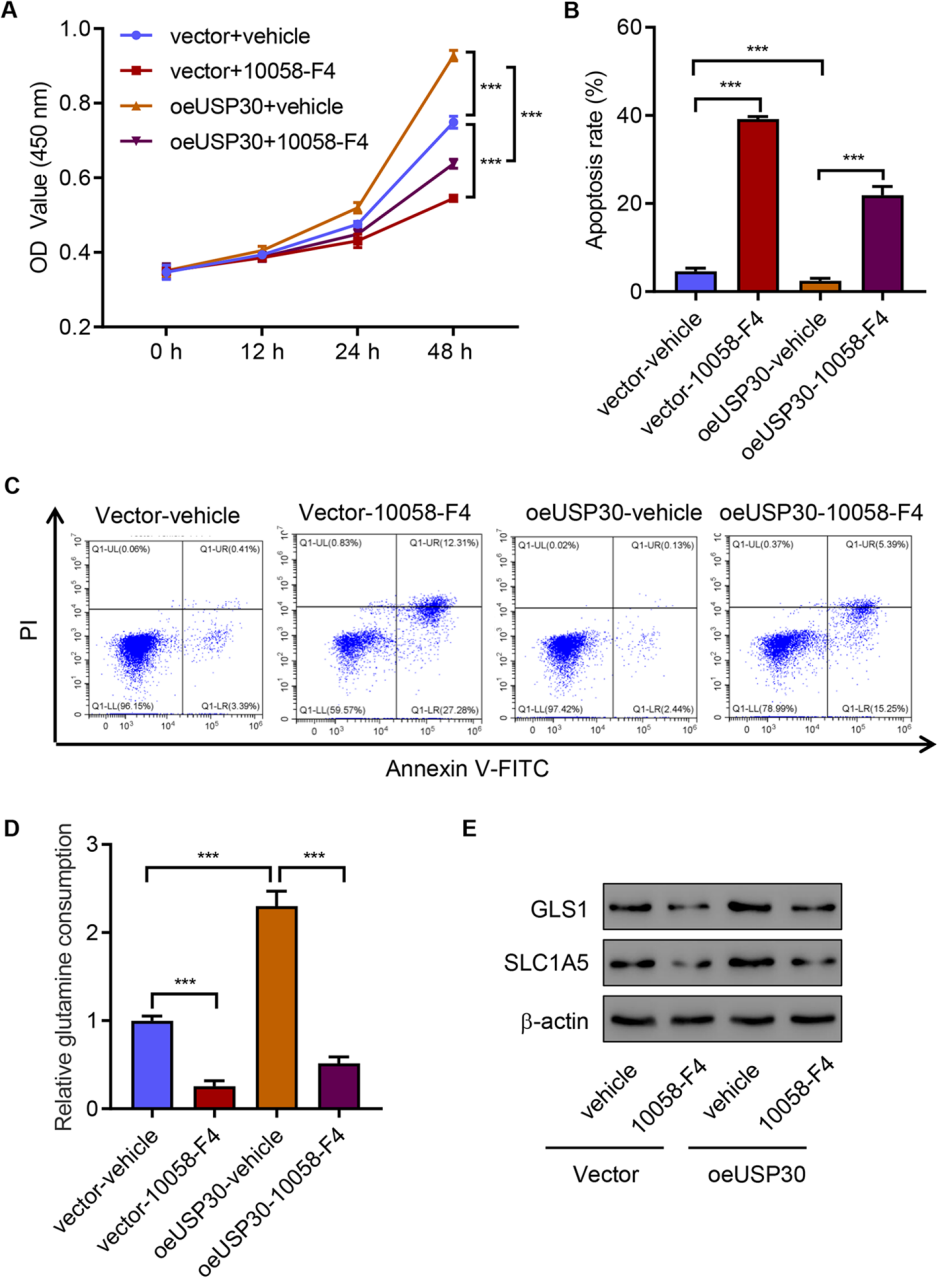
USP30 overexpression promotes SCC4 cell viability and glutamine consumption, and inhibits apoptosis through upregulation of c-Myc. **A** Cell viability, **B**, **C** apoptosis, **D** glutamine consumption, and **E** expression of GLS1 and SLC1A5 of SCC4 cells with USP30 overexpression and c-Myc inhibitor 10,058-F4 treatment. ***P < 0.001

To further study the role of USP30, we measured the effect of USP30 overexpression on cell viability, apoptosis, and glutamine consumption. The results showed that overexpressing USP30 promoted viability, suppressed apoptosis, and increased glutamine consumption (Fig. 4A–E). However, the c-Myc inhibitor 10058-F4 not only inhibited cell viability, promoted cell apoptosis, and decreased glutamine consumption, but it also ameliorated USP30 overexpression effects (Fig. 4A–E). All these findings indicate that USP30 overexpression promotes SCC4 cell viability and glutamine consumption, and inhibits apoptosis through upregulation of c-Myc.

### Characterization of NPs

**Fig. 5:**
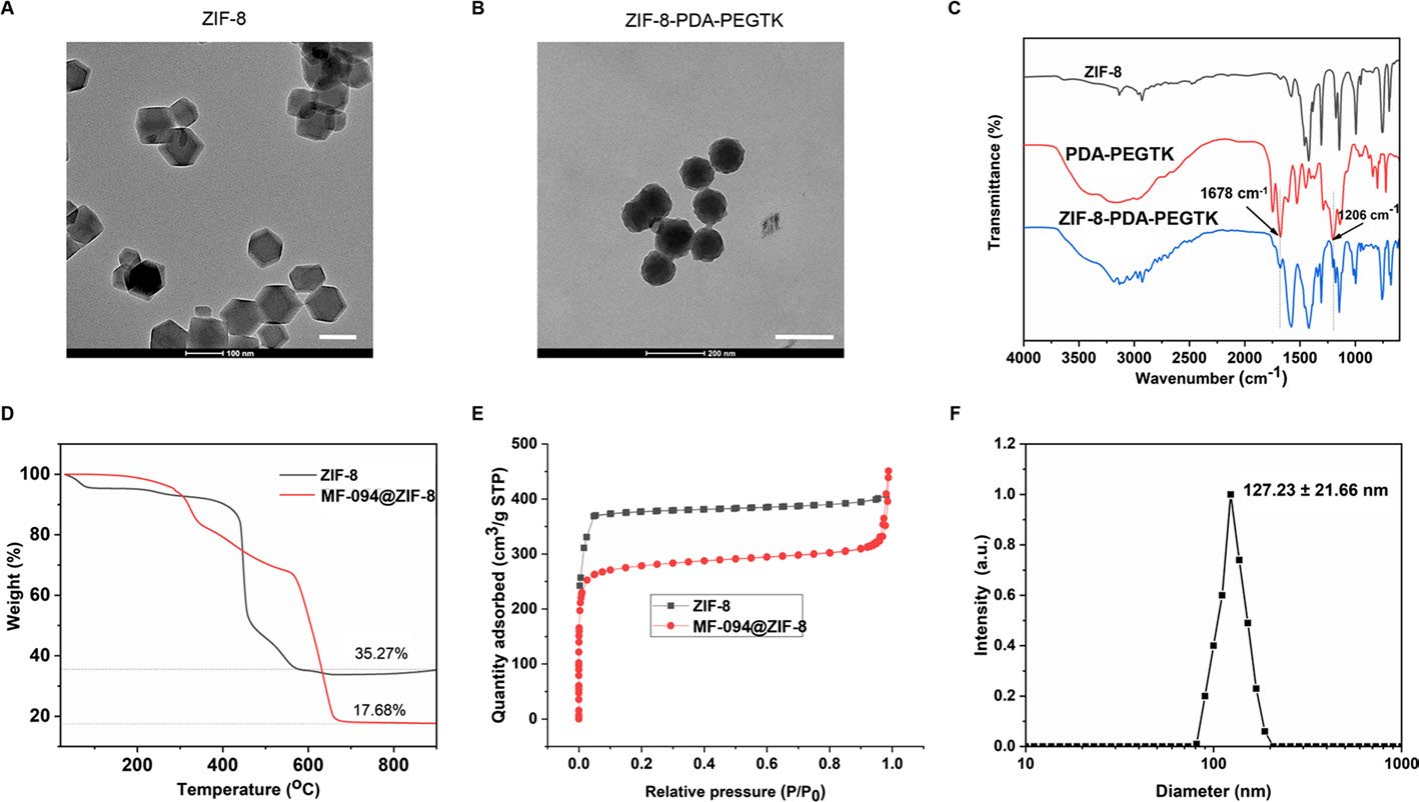
NP characterization. TEM images of (**A** scale bars: 100 nm) ZIF-8 and (**B** scale bars: 200 nm) ZIF-8-PDA-PEGTK. **C** FT-IR images of ZIF-8, TKPDA, and ZIF-8-PDA-PEGTK. **D** TGA isotherms of ZIF-8 and MF-094@ZIF-8. **E** N_2_ adsorption–desorption isotherms of ZIF-8 and MF-094@ZIF-8. **F** Size distribution of ZIF-8-TKPEG-PDA

TEM images in Fig. 5A and B revealed the uniform polyhedron morphology. PDA-PEGTK decoration resulted in a rougher surface and increased diameter. FT-IR analysis showed that large amount of surfactant in the ZIF-8 offers the C–H vibration bands at 2850–2930 cm^−1^ and C–H deformation bands at approximately 1465 cm^−1^ (Fig. 5C). The ZIF-8-PDA-PEGTK shows the C–H mode at 1678 and 1206 cm^−1^ again, even after extraction, which should be attributed to the C–H bonds in PEGTK block, and means that they had been successfully introduced to the nanoparticles surface. The ZIF-8 and MF-094@ZIF-8 showed ca. 64.7% and 82.3% mass loss (200–800 °C) compared with parent ZIF-8, respectively (Fig. 5D), which indicates that about 17.6% MF-094 was absorbed into ZIF-8 NPs, suggesting its relatively high loading capacity. Both of them showed a typical type I N_2_ absorption–desorption isotherm (Fig. 5E). The BET surface area of ZIF-8 and MF-094@ZIF-8 was 1381 and 656 m^2^/g, respectively. The pore volumes of MF-094@ZIF-8 and ZIF-8 were 0.84 and 0.29 cm^3^/g, respectively. The size distribution of ZIF-8-FDA-PEGTK is provided in Fig. 5F, in which ZIF-8-PDA-PEGTK NPs exhibited a diameter of 127.23 ± 21.66 nm.

### NPs enhance the anticancer effects of MF-094 in HSC4 cells

**Fig. 6:**
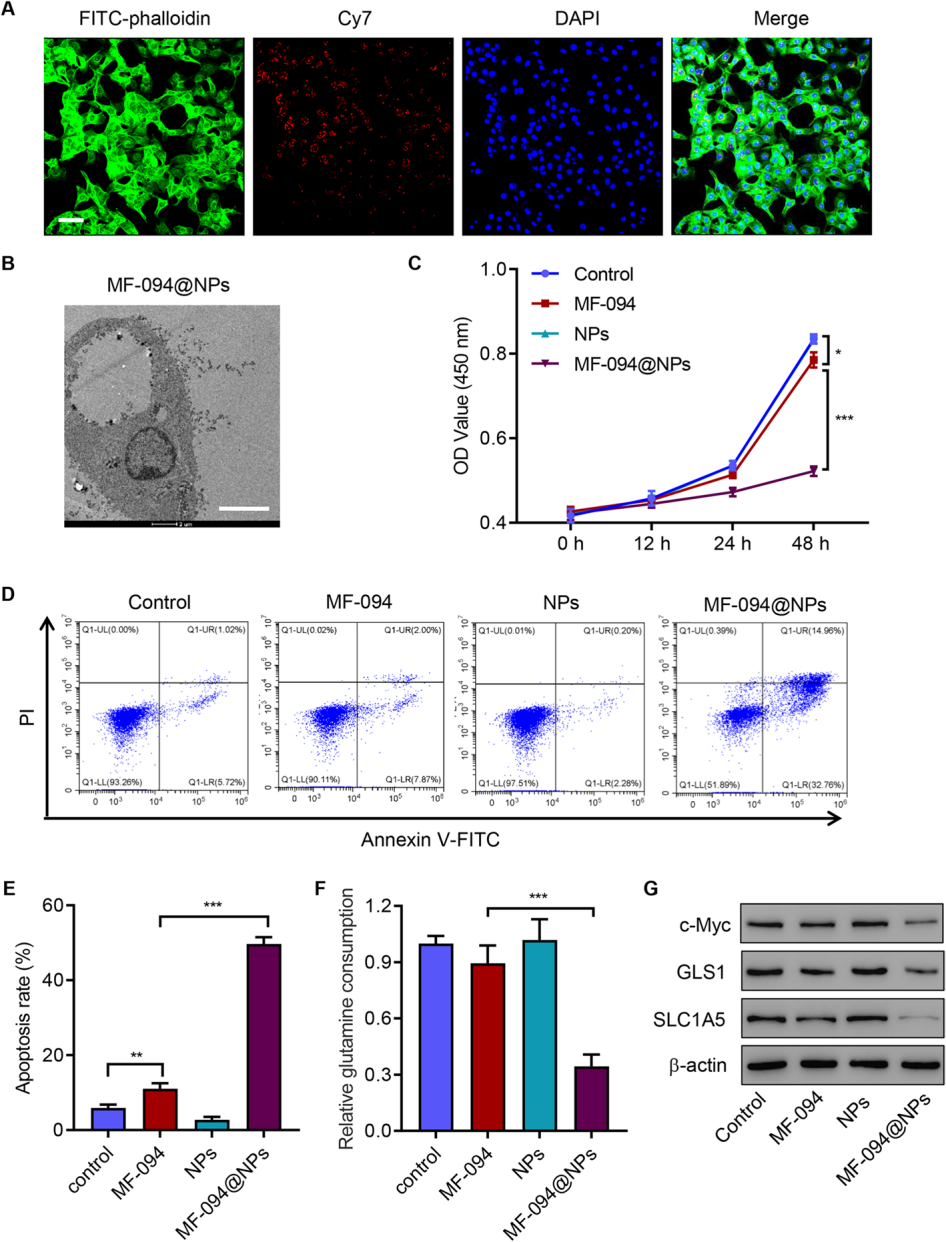
NPs enhance the anticancer effects of MF-094 in HSC4 cells. **A** CLSM images (scale bars: 50 μm). **B** TEM images showing intracellular distribution of MF-094@NPs in HSC4 cells (scale bars: 4 μm). **C** Cell viability, **D** and **E** apoptosis, **F** glutamine consumption, and **G** expression of c-Myc, GLS1, and SLC1A5 in HSC4 cells incubated with MF-094, NPs, and MF-094@NPs. *P < 0.05, **P < 0.01, ***P < 0.001

We then studied the effect of MF-094 and MF-094@ZIF-8-PDA-PEGTK (MF-094@NPs) on cell viability, and the results showed that MF-094 (from 0.2 to 2 μM) and MF-094@NPs (from 0.05 to 2 μM) significantly decreased the viability of HOEC and HSC4 cells, respectively, in a dose-dependent manner (Additional file 1: Fig S2A-S2B). The data suggest that MF-094@NPs enhanced the antitumor effect of MF-094. CLSM imaging showed that MF-094@NPs could be successfully taken up by HSC4 cells (Fig. 6A) and TEM images showed the cellular distribution of MF-094@NPs (Fig. 6B). Compared with MF-094, administration of MF-094@NPs significantly inhibited cell viability (Fig. 6C) and increased apoptosis (Fig. 6D and E). MF-094 treatment did not affect glutamine consumption, but MF-094@NPs treatment significantly decreased glutamine consumption (Fig. 6F). Western blotting results showed that MF-094@NPs treatment significantly decreased the expression of c-Myc, GLS1, and SLC1A5 compared with MF-094 (Fig. 6G). Together, the data demonstrate that NPs enhance the anticancer effects of MF-094 in HSC4 cells.

### Antitumor efficacy of MF-094@NPs in OSCC mouse model bearing SCC4 cells

**Fig. 7:**
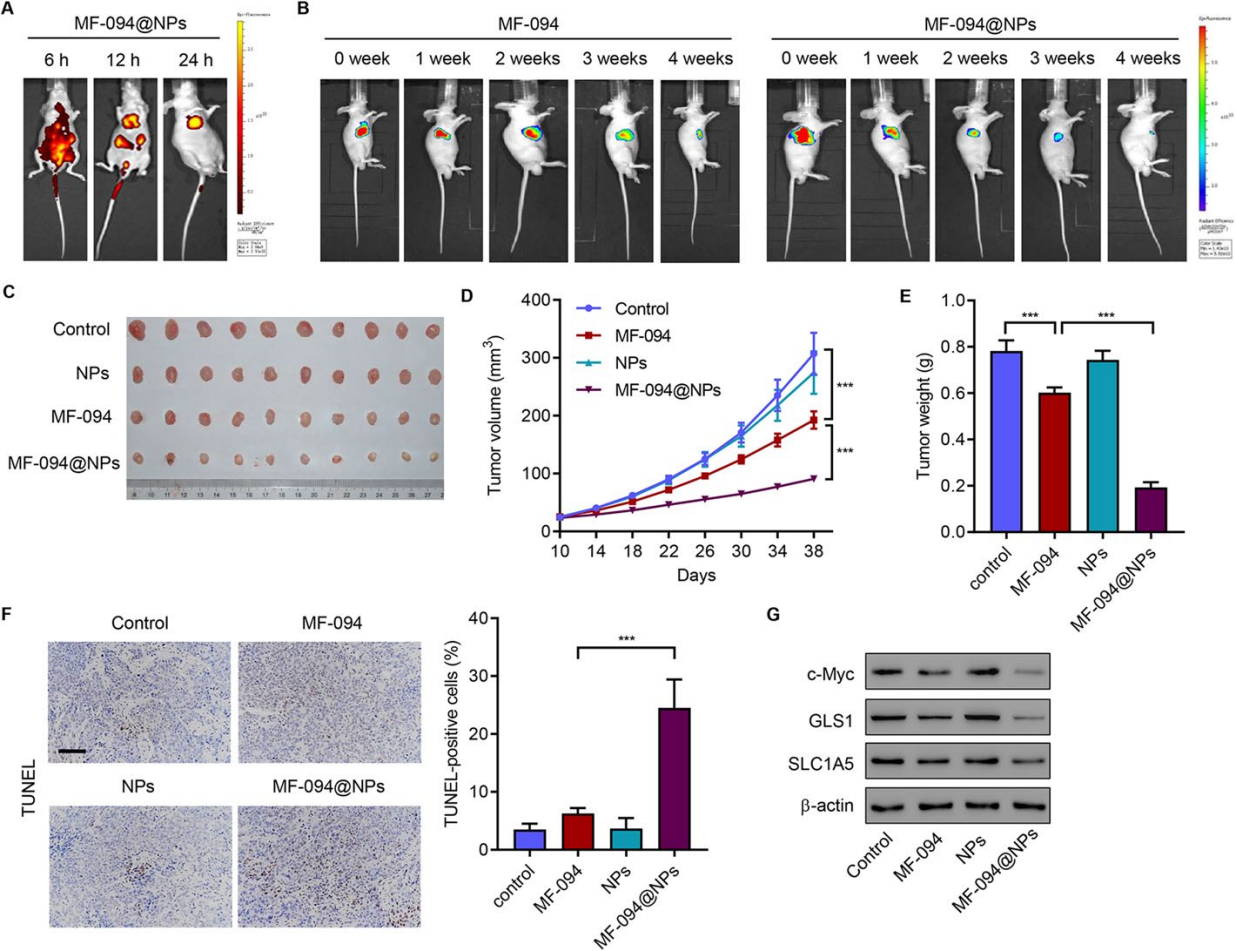
In vivo antitumor efficacy of MF-094@NPs. **A** Representative fluorescence (MF-094@NPs) and **B** bioluminescence (SCC4 cells) imaging of SCC4 cell subcutaneous injection-induced xenograft tumor model after intravenous MF-094 or MF-094@NPs injection (1 mg/kg/day). **C** Sample images of tumor among mice bearing SCC4 cells with different treatments. Tumor volume **D** and weight **E**. **F** TUNEL-positive cells were analyzed in different groups (scale bars: 100 μm). **G** Xenograft mouse tumors showing c-Myc, GLS1, and SLC1A5 expression. ***P < 0.001

To measure the antitumor effect of MF-094@NPs, SCC4 cells were inoculated into nude mice. Ten days after inoculation, Cy7-labeled NPs loaded with/without MF-094 were injected into the mice via the tail vein (1 mg/kg/day) for 4 weeks. NP fluorescent images were obtained using the IVIS 200 system at 6, 12, and 24 h to verify the NPs biodistribution (Fig. 7A). In vivo surviving SCC4 cells were detected using bioluminescence at 0, 1, 2, 3, and 4 weeks to verify the in vivo targeting efficacy of NPs (Fig. 7B). After euthanization, key organs (liver, heart, lungs, kidneys, and spleen) and xenografts were harvested, fixed, and processed to produce paraffin-embedded sections. NPs loaded with/without MF-094 had no toxicity in most organs (Additional file 1: Fig S3). Compared with MF-094, administration of MF-094@NPs significantly inhibited tumor growth (Fig. 7C), and decreased tumor volume (Fig. 7D) and tumor weight (Fig. 7E), but increased apoptosis (Fig. 7F). Western blotting results indicated that compared with MF-094, MF-094@NPs significantly decreased c-Myc, GLS1, and SLC1A5 (Fig. 7G). All the findings suggest that NPs enhance the anticancer effects of MF-094 in vivo.

## Discussion

In this study, we first analyzed USP30 expression in OSCC specimens, and the results showed that USP30 was significantly increased and its upregulation was associated with poor prognosis. Further analysis showed that USP30 overexpression significantly promoted cell viability and glutamine consumption, and inhibited apoptosis in OSCC cells. Study into the mechanism indicated that the effects of USP30 were mediated by deubiquitination of c-Myc. The results also support that targeting USP30 by MF-094@NPs significantly increased MF-094’s antitumor effect.

USPs have been implicated in different biological processes including cancer. For example, Madan et al. showed that the USP6 oncogene promotes Wnt signaling by deubiquitylating Frizzleds and inhibition of Wnt signaling significantly decreased the growth of USP6-driven xenograft tumors [20]. USP7 was significantly increased in hepatocellular carcinoma (HCC) and inhibition of USP7 efficiently suppressed tumor growth [21]. As one of the five types of DUBs, USP30 protects proteins from degradation by removing the ubiquitin [22]. USP30 inhibition has also been shown to promote aumdubin-induced apoptosis of lung cancer cells [9]. Our data indicate that the upregulation of USP30 was associated with poor prognosis and promoted OSCC progression.

c-Myc is one of the three members of the Myc family [23]. c-Myc is deregulated in many tumors; for example, c-Myc is increased in breast cancer and is related to elevated aggressiveness [24]. Studies have also shown that c-Myc cooperates with mutant β-catenin to drive hepatoblastoma [25]. In later stages of OSCC, c-Myc coordinates with other oncogenes to promote cancer progression [26]. We proved that USP30 promoted c-Myc deubiquitination to increase c-Myc protein levels. These findings revealed a new role of the USP30/c-Myc axis in OSCC, showing that USP30 suppressed c-Myc ubiquitination and degradation, leading to OSCC development.

Many current treatments for cancer are very toxic and have modest efficacy. Therefore, new therapeutics and targeted drug delivery are of great importance. NPs have circumvented limitations in the delivery of cancer therapeutics [27]. Jain et al. showed that NPs protected 5-fluorouracil against discharge to maintain the high local concentration and enhance its antitumor effect, but decrease its systemic toxicity [28]. Adhikari et al. indicated that drug-loaded ZIF-8 easily releases drugs under the acidic conditions of tumors [29]. Research has also indicated that when drugs with ZIF-8 are internalized, the ZIF-8 shell decomposes under acidic conditions, which allows the drug to be released in cancer cells, therefore increasing the anticancer effect [30]. All these studies suggest that MF-094-loaded ZIF-8 can be easily uptaken by cancer cells and then released, which will increase its anticancer efficiency. AA-PEG exosomes loaded with paclitaxel improved therapeutic effects in a pulmonary metastases mouse model [31]. In this study, the ZIF-8 nanoparticles were decorated with PDA-PEGTK to form ZIF-8-PDA-PEGTK. Our results showed that, compared with MF-094, administration of MF-094@NPs significantly inhibited tumor growth, suggesting that NPs enhance the anticancer effects of MF-094 in vivo. This might because the target identification of ZIF-8-PDA-PEGTK NPs results in more NPs being internalized, leading to high cytotoxicity for cancer cells [32]. There are certainly some limitations of this study. For instance, only one cell line was used for the in vivo study, and future studies using other types of OSCC cell lines should be carried out to validate the findings of this study. To further elucidate the function of USP30/c-Myc in OSCC, an orthotopic mouse model would be informative. Although setbacks exist, this study reveals a new mechanism underlying apoptosis of OSCC cells.

## Conclusions

USP30 regulated cell viability, glutamine consumption, and apoptosis in OSCC cells via regulating the deubiquitination of c-Myc. Targeting USP30 via a nanoparticle delivery system significantly increased its antitumor effect. This study highlights the importance of USP30/c-Myc signaling in OSCC, and may facilitate the development of therapeutics for OSCC.

## Supplementary Information

**Additional file 1.** Table S1. Clinicopathological characteristics and follow-up data of 72 patients with OSCC.

## Data Availability

All data presented in this study are included within the paper and its additional files.

## Abbreviations

OSCC: Oral squamous cell carcinoma
USPs: Ubiquitin-specific proteases
NPs: Nanoparticles
DUBs: Deubiquitinases
ZIF-8: Zeolitic imidazolate framework-8
HCC: Hepatocellular carcinoma
IHC: Immunohistochemistry
TEM: Transmission electron microscopy
BET: Brunauer–Emmett–Teller
TGA: Thermogravimetric analysis
FT-IR: Fourier transform infrared
CLSM: Confocal laser scanning microscopy
Co-IP: Coimmunoprecipitation
CHX: Cycloheximide
