# Supplementary material for: MF-094 nanodelivery inhibits oral squamous cell carcinoma by targeting USP30
Source: Cell Mol Biol Lett. 2022 Dec 6;27:107. doi: 10.1186/s11658-022-00407-8 (PMC9724415; doi:10.1186/s11658-022-00407-8)
Supplement: Supplementary file 1 — Additional file 1. Table S1. Clinicopathological characteristics and follow-up data of 72 patients with OSCC. [file 11658_2022_407_MOESM1_ESM.docx]

**Table S1.** Clinicopathological characteristics and follow-up data of 72 patients with OSCC

| Characteristics | USP30 expression | | *P* value |
| --- | --- | --- | --- |
|  | Low | High |  |
| **Gender** |  |  | 0.915 |
| Male (n=32) | 14 | 18 |  |
| Female (n=40) | 17 | 23 |  |
| **Age (years)** |  |  | 0.059 |
| <50 (n=26) | 15 | 11 |  |
| ≥50 (n=46) | 16 | 30 |  |
| **TNM stage** |  |  | 0.014 |
| I+ II (n=30) | 18 | 12 |  |
| III (n=42) | 13 | 29 |  |
| **Lymph node metastasis**  Positive (n=41)  Negative (n=31) | 12  19 | 29  12 | 0.007 |

Differences between groups were determined by the Chi-square test.


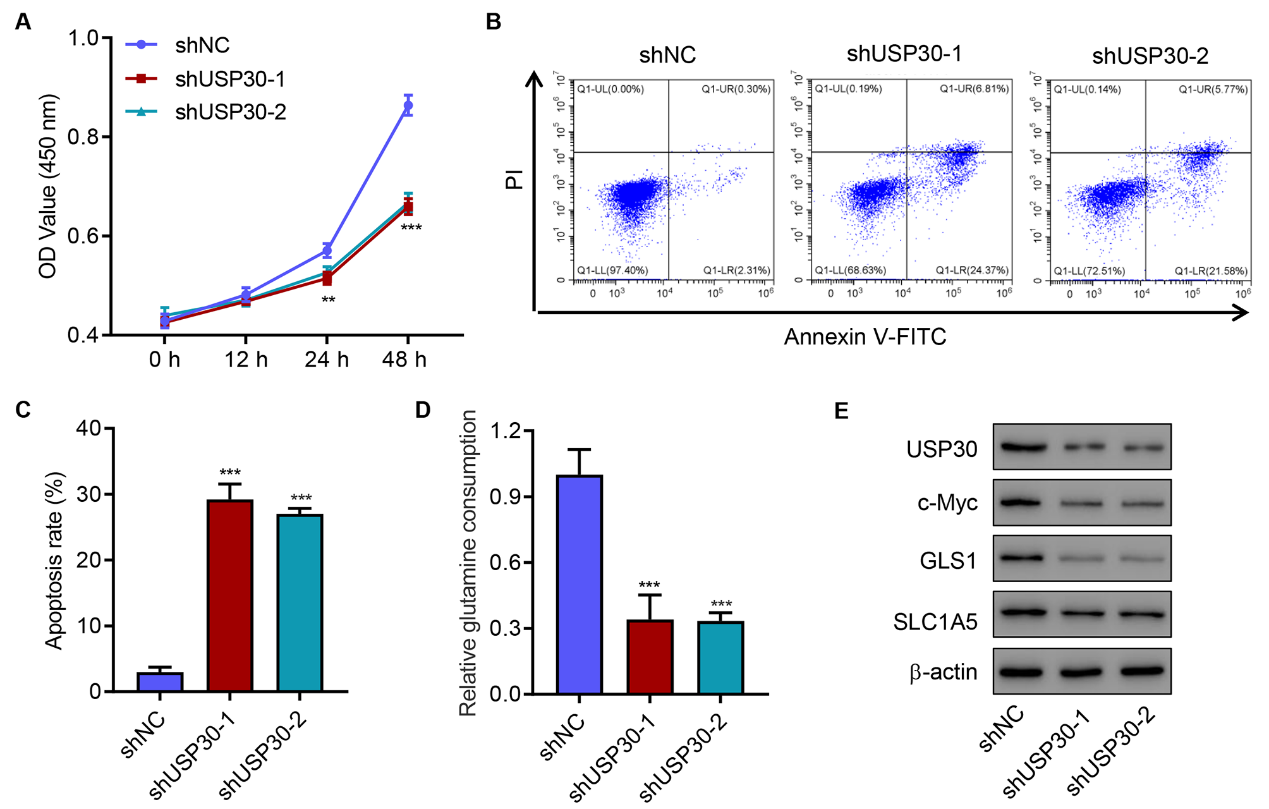


**Figure S1.** USP30 knockdown inhibits HSC4 cell viability, glutamine consumption and induces apoptosis *in vitro*. (A) Cell viability, (B, C) apoptosis, (D) glutamine consumption and (E) expression of USP30, c-myc, GLS1 and SLC1A5 in HSC4 cells with or without USP30 knockdown. ***P* < 0.01, ****P* < 0.001 vs shNC.


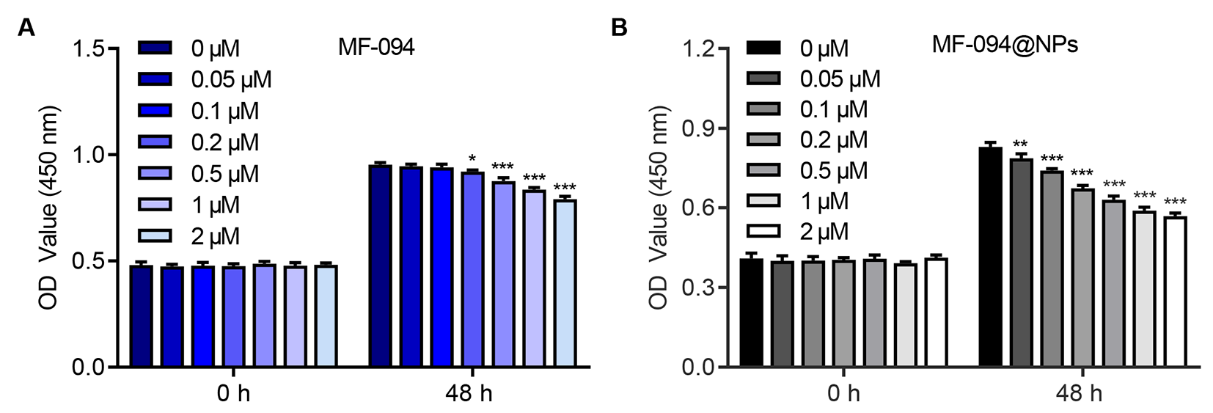


**Figure S2.** Cell viability of (A) HOEC and (B) HSC4 cells incubated with different concentrations of USP30 inhibitor MF-094 and MF-094@NPs for 0 and 48 h, respectively. **P* < 0.05, ***P* < 0.01, ****P* < 0.001 vs 0 μM.


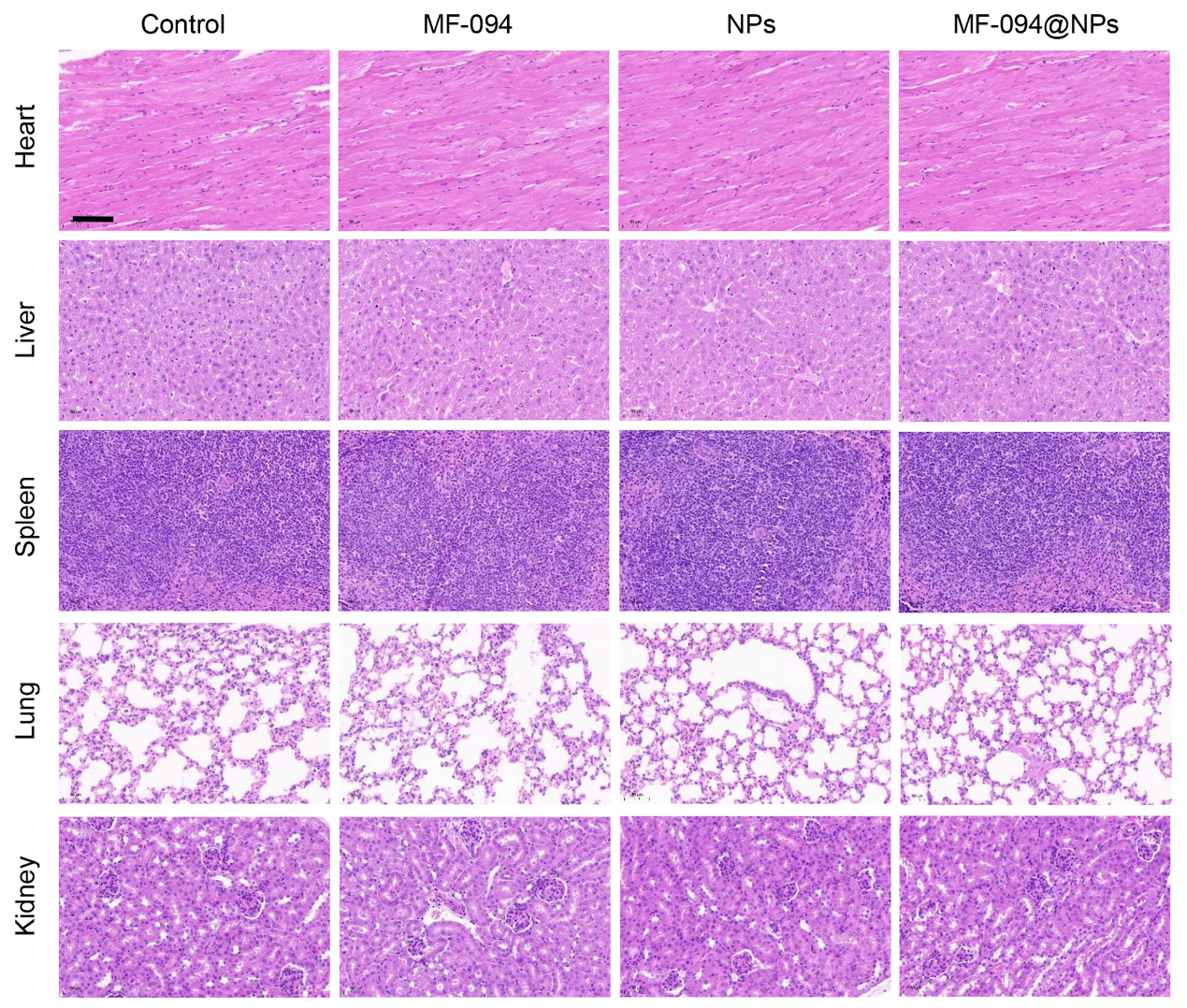


**Figure S3.** Images of H&E-stained sections obtained from key organs (liver, heart, spleen, kidneys, and lungs) from xenograft mice at 4 weeks post-injection (Scale bars: 100 μm).
